# Josephson dynamics and Shapiro steps at high transmissions: current bias regime

**DOI:** 10.3762/bjnano.15.5

**Published:** 2024-01-11

**Authors:** Artem V Galaktionov, Andrei D Zaikin

**Affiliations:** 1 I.E. Tamm Department of Theoretical Physics, P.N. Lebedev Physical Institute, 119991 Moscow, Russia; 2 National Research University Higher School of Economics, 101000 Moscow, Russia

**Keywords:** dissipation, Josephson effect, Shapiro steps, superconducting junctions

## Abstract

We investigate Josephson dynamics of highly transparent superconducting nanojunctions at subgap voltages and temperatures. In this limit, intrinsic dissipation in such junctions turns out to be sub-Ohmic, which yields a linear dependence of the average voltage on the bias current *I* slightly exceeding the critical one *I*_c_. We demonstrate a strong impact of intrinsic sub-Ohmic dissipation on integer Shapiro steps appearing on the *I*–*V* curve in the presence of external microwave radiation.

## Introduction

The key signature of the ac Josephson effect in superconducting junctions is the presence of coherent current oscillations with the fundamental frequency ω_J_ = 2 *eV*/ℏ, where *V* is the voltage applied to the junction and −*e* is the electron charge. Under the influence of external microwave radiation with frequency ω, current jumps appear on the junction *I*–*V* curve, which are known as Shapiro steps [1]. The presence of such steps is the result of a resonance between Josephson oscillations and the external microwave signal. In tunnel junctions, the primary resonance occurs under the condition ω = ω_J_. In a more general case, the corresponding condition takes the form


[1]

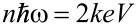



or, equivalently, ω = (*k*/*n*)ω_J_, where *k* and *n* are positive integer numbers. The values *k* ≥ 2 correspond to the presence of higher harmonics of the Josephson current emerging because of a possibly non-sinusoidal current–phase relation, whereas the numbers *n* ≥ 2 account for multiphoton processes, which may become non-negligible at higher amplitudes of the microwave signal and/or at smaller frequencies ω. One can distinguish integer and fractional Shapiro steps corresponding to, respectively, integer and non-integer values of the ratio *k*/*n*.

Note that resonances leading to Shapiro steps on the *I*–*V* curve occur not only in the limit of bias voltages *V* that are constant in time, but also, for example, in the current bias limit, that is, when the current across the system is externally fixed. Obviously, in the latter case, the voltage *V* cannot remain constant in time anymore, and the condition in Equation 1 should be modified by replacing *V* by its time average *V*→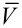.

Dissipation usually plays an important role in the case of current-biased superconducting nanojunctions. One possible way to account for dissipative currents is to employ the so-called resistively shunted junction (RSJ) model [1]. In the case of tunnel junctions, this phenomenological model can be microscopically justified only at temperatures in the vicinity of the critical temperature *T*_c_. As one goes away from *T*_c_, the number of quasiparticles above the superconducting gap decreases exponentially and, hence, no dissipative currents at subgap voltages and *T* → 0 can flow across the junction.

The situation becomes entirely different provided one goes beyond the tunneling limit and considers highly transparent superconducting weak links in which case the charge transfer is essentially controlled by the mechanism of multiple Andreev reflection [2]. This mechanism causes intrinsic dissipation at subgap energies. Recently, we demonstrated [3] that such intrinsic dissipation has a dominating sub-Ohmic component in the subgap regime. This observation implies, for example, substantial modifications of the *I*–*V* curve at bias currents *I* just slightly exceeding the critical value *I*_c_. In particular, one finds [3] 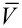 ∝ *I* − *I*_c_ instead of the square root dependence 
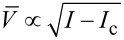
 derived within the standard RSJ model.

In this work we point out that sub-Ohmic subgap dissipation in transparent superconducting nanojunctions may substantially modify the whole pattern of integer Shapiro steps as compared to that observed in the Ohmic limit.

## Results and Discussion

Below, we are going to consider a purely ballistic SNS nanojunction with 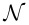 fully transparent conducting channels and normal state conductance 1/*R*_N_ = 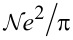. The thickness of a normal (N) layer *d* between two superconducting (S) electrodes is kept much shorter than the superconducting coherence length, that is, *d* ≪ ξ_0_ ∼ *v*_F_/Δ. Here, Δ is the absolute value of the order parameter in superconducting electrodes and *v*_F_ denotes the Fermi velocity. For simplicity here and below, we set the Planck and Boltzmann constants equal to unity (ℏ = *k*_B_ = 1).

In what follows, we will first re-derive the dc *I*–*V* curve for our ballistic nanojunction in the current bias regime. In this part of our analysis, we will follow closely our recent publication [3]. Then, we will add an ac current signal and recover the expressions for Shapiro steps in the system under consideration.

Biasing the junction by a voltage *V* that is constant in time, one induces the current *I*(*t*) across this junction, which can be cast to the form of the Fourier series


[2]

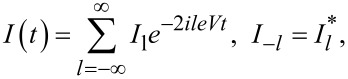



where the general expressions for all current harmonics *I*_l_ have been derived microscopically [4–5]. We are interested in the limit of small bias voltages *eV* ≪ Δ and low temperatures *T* ≪ Δ, where one finds [3]


[3]

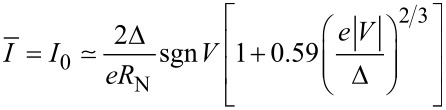



and


[4]

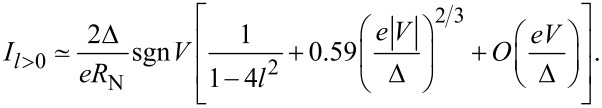



Note that the expression for the second term in the square brackets in Equation 4 holds only for *l* ≪ Δ/*e*|*V*|, while the last term in this equation is not specified since it remains parametrically small as long as the inequality *eV* ≪ Δ is satisfied. It is worth pointing out, however, that in order to disregard this term for sufficiently large numbers *l*, it would be necessary to additionally verify that the omitted terms do not grow with *l*; otherwise this approximation will fail for large enough *l*.

Let us now focus our attention on the current bias regime, that is, let us assume that a current *I* that is constant in time flows across our SNS nanojunction. Clearly, in this case the voltage *V* cannot anymore remain independent of time, and the applicability of the above Equations 2–4 needs to be reconsidered. Recently, it was demonstrated [3] that provided the voltage changes adiabatically and remains small enough, that is, *V*(*t*) ≪ Δ/*e*, the voltage dynamics in the current bias regime can be described by a simple equation,


[5]

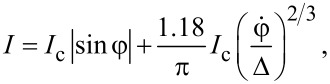



where *I*_c_ = πΔ/(*eR*_N_) is the critical current of our weak link at *T* → 0 and φ(*t*) equals to one half of the Josephson phase being related to the voltage *V*(*t*) across the junction by means of the standard Josephson relation 
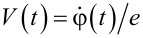
.

In order to solve this equation, it will be convenient for us to introduce a dimensionless variable,


[6]

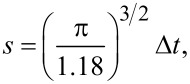



thereby reducing Equation 5 to


[7]

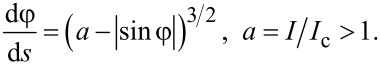



The solution φ_0_(*s*) of the latter equation can be written in the form


[8]

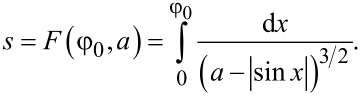



For 0 ≤ φ_0_ ≤ π, we obtain


[9]

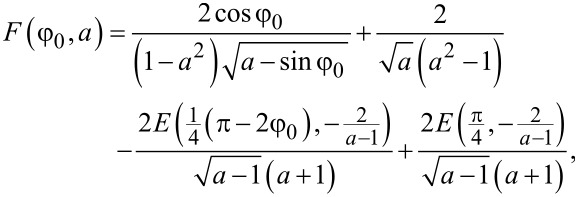



where 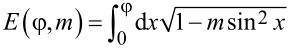 is an incomplete elliptic integral. For larger φ_0_ in the interval *p*π *<* φ_0_ *<* (*p* + 1)π with integer *p*, one has


[10]

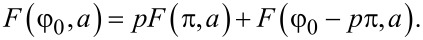



**Figure 1:**
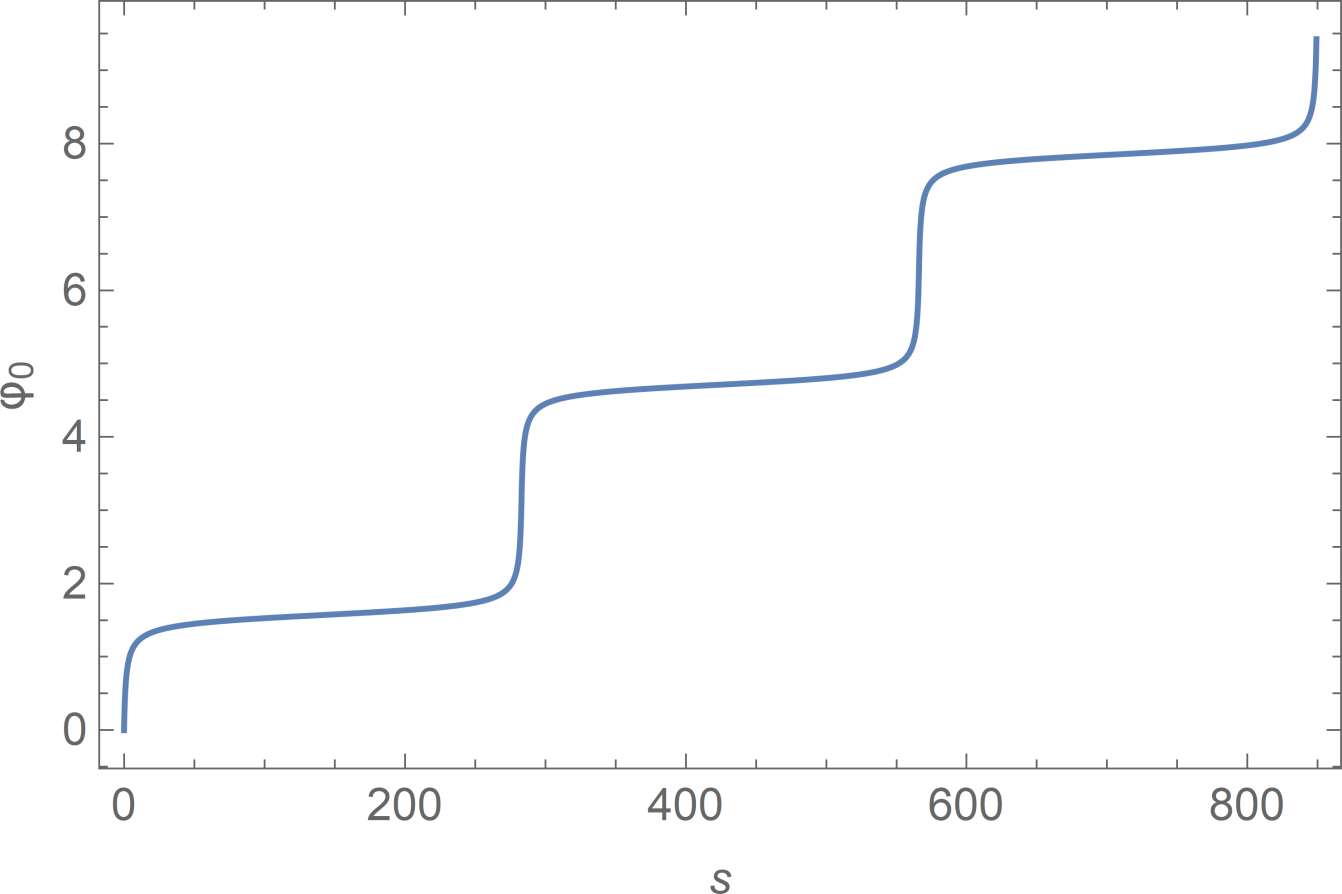
The solution of Equation 7 φ_0_(*s*) evaluated at *a* = 1.01.

The solution φ_0_(*s*) (Equation 8) of Equation 7 is also displayed in Figure 1.

Employing the Josephson relation between the voltage and the phase variables and averaging the resulting expression for *V*(*t*) over time, we immediately recover the *I*–*V* curve in the form [3]


[11]

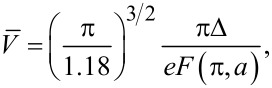



where


[12]

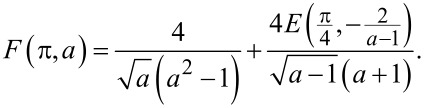



In the limit *a* − 1 ≪ 1, that is, provided the current *I* just slightly exceeds *I*_c_, this result reduces to a simple formula


[13]

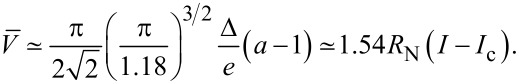



Verifying the assumption adopted in the beginning of our calculation, we observe that the average voltage (Equation 13) obviously obeys the condition 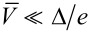 in the limit *I* − *I*_c_ ≪ *I*_c_, which is interesting for us. The same is true for the instantaneous voltage values *V*(*t*), which remain small for most of the time, raising up to *V*(*t*) ∼ Δ/*e* (implying the borderline of applicability of our calculation) only within short (in the measure of *a* − 1 ≪ 1) time intervals in the immediate vicinity of the phase values φ = π*m* (with *m* = 0, ±1, ±2, …), where the current component *I*_c_|sinφ| tends to zero. It is easy to check that the presence of such sharp voltage peaks can by no means alter any of our results and conclusions and may at most lead to an insignificant modification (decrease) of the numerical prefactor on the right-hand side of Equation 13.

The result (Equation 13) demonstrates that the *I*–*V* curve of fully transparent superconducting junctions is expected to be linear in the current bias regime as long as the condition *I* − *I*_c_ ≪ *I*_c_ remains satisfied. These expectations appear to be supported by several recent experiments [6–8] performed with different types of transparent superconducting junctions (see also [3] for more details on the comparison between theory and experiment).

In contrast, the dependence 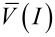 (Equation 13) clearly differs from the square root one 
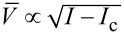
, which is typical for the RSJ model [1] and was also derived for transparent superconducting weak links subject to Ohmic dissipation produced by an external shunt resistor [9]. Hence, the *I*–*V* curve in highly transparent superconducting weak links in the current bias regime and at sufficiently low voltages may significantly depend on the form of a leading dissipative contribution to the current. More generally, replacing the last term on the right-hand side of Equation 5 by 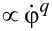 and proceeding in much the same way as above, we arrive at the dependence 
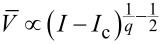
, which embraces both our result (Equation 13) and the square root dependence [9] derived in the Ohmic limit.

Let us now – in addition to the constant current component *I* – bias our junction by an ac component, which amounts to replace


[14]

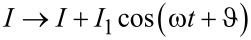



on the left-hand side of Equation 5. Here, ω is the frequency of the ac signal and ϑ is an arbitrary phase changing from −π to π. Again introducing the dimensionless variable (Equation 6) and slightly generalizing our approach in order to include an arbitrary sub-Ohmic dissipation 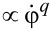, we have


[15]

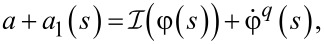



where


[16]

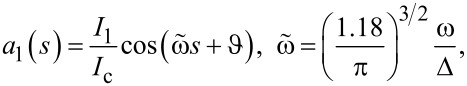




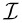
(φ) is an arbitrary π-periodic function, and we assume *q* ≤ 1. Provided *a*_1_ → 0, 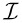 = |sinφ| and *q* = 2/3 Equation 15 reduces to Equation 7.

Equation 15 allows one to consider both sub-Ohmic and Ohmic dissipation on equal footing. The latter sets in either in the presence of an external shunting resistance or in the regime of higher voltages 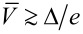, in which case the current–phase relation deviates substantially from *I* = *I*_c_|sinφ|.

Let us assume that the amplitude of the ac signal *I*_1_ is small compared to *I*_c_, and in addition to this, *a*_1_(*s*) ≪ *a*, implying that the term *a*_1_(*s*) in Equation 15 can be treated as a perturbation. Then the solution of this equation can be expressed in the form


[17]

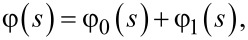



where, as before, the solution of the unperturbed Equation 15 with *a*_1_(*s*) = 0 is denoted as φ_0_(*s*), while φ_1_(*s*) represents the correction of the first order in *a*_1_ to it. Expanding Equation 15 up to the first order in φ_1_, we get


[18]

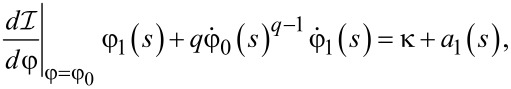



where κ = δ*I*/*I*_c_ and δ*I* is the correction to the constant current *I* due to the presence of an ac signal. Taking the derivative of Equation 15 with respect to *s* at *a*_1_ = 0, we obtain


[19]

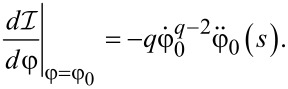



Substituting this expression into Equation 18, we arrive at the following equation


[20]

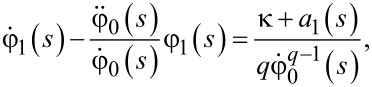



which can be resolved in a straightforward manner with the result


[21]

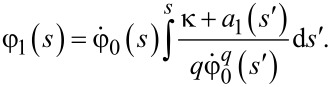



This expression defines the correction to both the Josephson phase and the voltage across the junction, provided the voltage dynamics in the absence of the ac signal is known.

The time derivative of the phase φ_1_ (Equation 21) defines an extra voltage generated by an ac current signal. Verifying that the time average of this extra voltage equals to zero [10], we observe that it is the case at all frequencies 
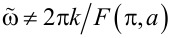
, implying that the current correction δ*I* = 0, and we get back to the *I*–*V* curve (Equation 11) derived in the absence of an ac signal. The non-zero value of the correction to the current that is constant in time, δ*I* ≡ κ*I*_c_, occurs provided 
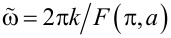
 or, equivalently, at frequencies 
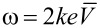
. In this case we arrive at the condition


[22]

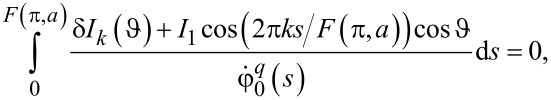



which determines the magnitude of the current correction δ*I_k_*(ϑ) for all integer values of *k*.

We consider the limit of small voltages and sub-Ohmic dissipation with *q* = 2/3, which is interesting for us. Combining Equation 22 and Equation 12, we obtain


[23]

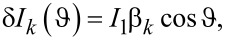



where β*_k_* are numerical prefactors independent of *a*. Their values can be determined numerically with the aid of Equation 22. For instance, for *k* = 1,…, 5, we get


[24]

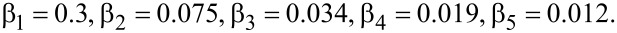



The total magnitude of the corresponding Shapiro step is defined by the formula


[25]

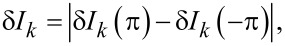



which obviously yields δ*I_k_* = 2*I*_1_β*_k_*.

For comparison, let us also consider the limit of Ohmic dissipation, that is, we now set *q* = 1. In this case, we have


[26]

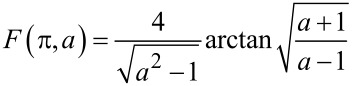



and from Equation 22, we immediately recover the result [11] for δ*I_k_*(ϑ), which in the limit *a* − 1 ≪ 1 reduces to


[27]

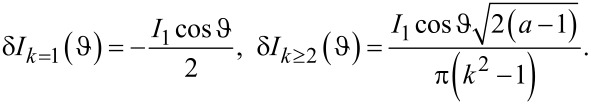



The magnitudes of the Shapiro steps are again defined by combining Equation 27 and Equation 25.

## Conclusion

**Figure 2:**
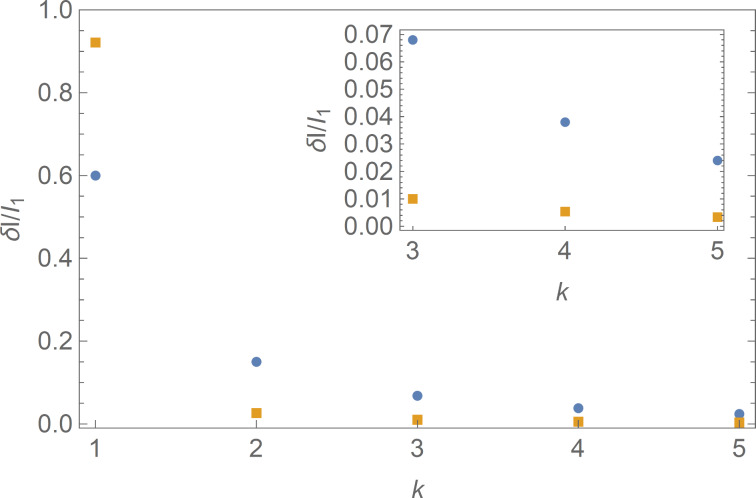
The magnitude of Shapiro steps δ*I_k_* evaluated with the aid of Equation 22 and Equation 25 for sub-Ohmic (blue circles) and Ohmic (orange squares) dissipation at *a* = 1.01. Inset: the same steps for *k* = 3, 4, and 5 zoomed for clarity.

Comparing the magnitudes of Shapiro steps in the sub-Ohmic and Ohmic limits for different values of *k*, we observe that the first Shapiro step 2*I*_1_β_1_ in the sub-Ohmic limit turns out to be smaller than that in the Ohmic limit, cf. also Equation 27. In the Ohmic limit, the amplitudes of all Shapiro steps with *k* ≥ 2 contain an extra parametrically small factor 
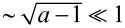
, which is absent in the sub-Ohmic case (Equation 23, Equation 24). In other words, for *I* − *I*_c_ ≪ *I*_c_, Ohmic dissipation yields strong suppression of all integer Shapiro steps except for the one with *k* = 1, as if one would deal with standard tunnel junctions described by a purely sinusoidal current–phase relation. In contrast, all integer Shapiro steps survive (with amplitudes slowly decreasing with growing *k*) in the case of sub-Ohmic dissipation, thereby illustrating an essentially non-sinusoidal current–phase relation featuring highly transparent superconducting weak links. Such behavior of Shapiro steps is illustrated in Figure 2.

As we already pointed out in the Introduction, at sufficiently high amplitudes of an ac signal, one can also observe fractional Shapiro steps at frequencies ω = ω_J_/2, ω =ω_J_/3, and so on. In the case of highly transparent superconducting nanojunctions with predominantly Ohmic dissipation, such fractional steps can also become important and, under certain conditions, they may even dominate over the integer steps [11]. However, it is easy to see that, in the case of sub-Ohmic dissipation considered here, fractional Shapiro steps remain negligible in the current bias limit. In contrast, in the voltage bias limit, fractional steps may remain significant also in the sub-Ohmic case being described by essentially the same relations as in [11] with only small corrections, ∼(*eV*/Δ)^2/3^ ≪ 1. This observation concludes our analysis of Shapiro steps in highly transparent superconducting weak links.
